# COVID-19 related lockdown: a trigger from the pre-melancholic phase to catatonia and depression, a case report of a 59 year-old man

**DOI:** 10.1186/s12888-020-02978-2

**Published:** 2020-11-25

**Authors:** Giuseppe Sarli, Lorenzo Polidori, David Lester, Maurizio Pompili

**Affiliations:** 1 Psychiatry Residency Training Program, Faculty of Medicine and Psychology, Sapienza University of Rome, Rome, Italy; 2 Department of Psychology, Stockton University, Galloway, NJ USA; 3 Department of Neurosciences, Mental Health and Sensory Organs, Faculty of Medicine and Psychology, Suicide Prevention Centre, Sant’Andrea Hospital, Sapienza University of Rome, Rome, Italy

**Keywords:** Lockdown, Typus Melancholicus, Catatonia, Major depressive disorder, Covid-19

## Abstract

**Background:**

The pre-melancholic model described by Tellenbach may provide a common model for understanding the psychological implications of the lockdown. In this case report, we describe a rare catatonic status as a psychological implication linked to the COVID-19 pandemic, a really unique global situation.

**Case presentation:**

B is a 59 year-old man with mute psychiatric anamnesis whose mother suffered from a major depressive disorder. As the lockdown began, he started to develop concerns about his family’s economic condition. According to his wife, he could see no end to the epidemic and no future at all. Moving from this, he started to show a severe and rapidly progressive depression and to develop mood congruent delusions. In addition, he had increasing anhedonia, apathy, starvation and insomnia. This turned in the end into a catatonic-like state, along with a deep desire to die.

Admitted to the psychiatry ward in a state of mutism, he was discharged after 15 days with a diagnosis of “Major depressive disorder, single severe episode with no psychotic behavior”. He was treated with Sertraline, Olanzapine and Lorazepam.

**Conclusions:**

Our aim is to draw attention to the effect of the lockdown upon a Tellenbach-like personality structure. Identifying this type of pre-morbid personality structure could help clinicians understand and treat some cases of patients with severe major depressive disorders elicited by the COVID-19 pandemic.

## Background

In December 2019, an unexpected epidemic broke out in in Wuhan, Hubei Province, China [1]. A novel coronavirus was identified and found to be responsible for a disease named by the World Health Organization as coronavirus disease 2019 (COVID-19). COVID-19 is an acute respiratory syndrome caused by the coronavirus 2 (SARS-CoV-2) [2]. So far, the virus has caused more than 400,000 deaths, affecting almost 8 million people in 229 countries [3]. Italy has been one of the most hard hit countries, with a peak in March 2020. On March the 11th, the Istituto Superiore di Sanità confirmed that Italy was second in the number of deaths due to COVID-2019 after China [4]. On the same day, the whole country was declared a red zone, and a lockdown began. The Italian Government introduced progressive mitigation measures that ended with the mandatory closing of almost all commercial and productive activities in order to drastically limit social interaction and prevent the spreading of the virus [5–7].

The pandemic brought, not only the risk of death because of the infectious disease, but also psychological suffering and discomfort as a result of the risk of being infected by the virus and the great socioeconomic changes linked to the quarantine (such as social distancing, financial problems and changes in routines) [8]. These stressors were experienced by patients with a pre-existing psychiatric disease, but also by those with no psychatric history, thereby resulting in new psychiatric problems [9, 10].

Below, we describe the case of an adult with no prior psychiatric history, who developed catatonia and showed severe depressive symptoms after facing economic struggles and quarantine isolation during the COVID-19 pandemic.

## Case presentation

B is a 59 year-old man living in Rome with his wife, with no prior psychiatric history but whose mother had been treated by a specialist for a major depressive disorder. The couple lives in a residential suburban area of the Italian capital, and they both come from middle-low income families. B does not have any brothers or sisters, and he inherited the house where the couple lives. They got married after many years in a relationship, and they do not have any children. The couple runs a Shiatsu center that is their only source of income. They have a professional staff working with them, and this, in addition to the bills and the rent, makes the management costs of their business high. Thus, they have to work long hours in order to preserve their economic stability. They are both shy, and they have few friends.

B is described by his wife as a meticulous person with a strong sense of duty, concerned with orderliness and very emotional. His concern with orderliness affects his interpersonal relationships, especially with his family members. He finds it very difficult to express disagreement with his parents and with his wife. His greatest concern is his wife’s satisfaction and happiness in order to maintain harmony in their relationship, and this manifests itself in his work. For him, there is peace and serenity when things occur as expected. When unpredicted events occur, he becomes anxious and tries to resume his life as planned.

His sense of duty is illustrated by his way of relating to other people. For him, it is crucial to not do anything wrong and always to be correct and not in debt to others. At work, his main goal is to keep his clients satisfied and avoid any quarrel with them. If any problem occurs, he feels guilty, and he focuses on solving the problem.

All these pre-morbid elements belong to the *Typus Melancholicus* described by the German psychiatrist Hubertus Tellenbach (1914–1994) as a type of personality related to unipolar depression [11, 12].

On March 11th, they were obliged to close their center as a consequence of the COVID-19 lockdown. B became very concerned about their economic situation and, as reported by his wife, he could see no end to this epidemic and no future opportunity to restart their business and overcome COVID-19 related economic issues. He began to show a severe and rapidly progressive depression and to develop mood-congruent delusions related to pauperization and ruin, as well as hopelessness, anhedonia, apathy, starvation and insomnia. In the 10 days preceding his hospitalization, he slept only a few minutes each day, and he did not eat at all. Furthermore, his wife reported that the patient seemed unable to perceive and reply to environmental stimuli. The only words he uttered were about his hopelessness, ruin and financial crisis, and he was unable to see himself in the future. He had undergone a narrowing of the field of consciousness, as described by Janet in 1909, in both sensory perception and behavior [13], in his case, focused on financial problems related to COVID-19.

In the 2–3 days prior to his admission, he was reported as lying on the bed all day in a catatonic-like state, without speaking or replying to his wife. The only thought he was able to verbalize was his desire to die. He begged his wife to end his life, which led her to call an ambulance.

After his admission to the emergency room, he remained in a state of mutism. He would shake his head to reply to the psychiatrist’s questions. When he was asked by the doctor, “Do you think I can help you?” he shook his head to indicate “No” and moved his eyes. As listed in the Diagnostic and Statistical Manual of Mental Disorders Fifth Edition, there were three or more symptoms in order to diagnose catatonia [14]; more specifically, B displayed stupor, mutism and waxy flexibility. He cooperated for the nasopharyngeal swab to tested for COVID-19. A brain CAT scan indicated no organic lesions. A 4 mg Lorazepam vial and a 5 mg Olanzapine tablet were administered. The patient was then moved to our psychiatry ward for further observation and treatment.

Once in our psychiatry ward, Mr. B was assessed with several psychometric scales. We used the Columbia – Suicide Severity Rating Scale (C-SSRS), the Physical and Psychological Pain Scale, the Orbach Mental Pain Scale (OMMP), the Psychache Scale (PAS) and the Beck Hopelessness Scale (BHS) in order to evaluate his suicide risk, ideation and intention. Moreover, we tested for the presence of anxious-depressive disorders using the Hamilton Rating Scale for Depression (HAM-D), the Beck Depressive Inventory (BDI-II) and the Hamilton Rating Scale for Anxiety (HAM-A). Furthermore, we administered the Brief Psychiatric Rating Scale (BPRS) for a global psychiatric evaluation of the patient, the Childood Trauma Questionnaire (CTQ) to assess any traumatic history, and the Insight Scale (IS) to measure his level of insight.

He was discharged after 15 days, during which he showed slow improvement. He was diagnosed with “Major depressive disorder, single severe episode with no psychotic behavior”, and the following therapy was prescribed: Sertraline 50 mg 2 tablets at 8.00; Olanzapine 5 mg 1½ tablet at 22.00 and Lorazepam 2.5 mg 1 tablet at 20.00.

## Discussion and conclusions

The depressive episode of B resembles the catamnestic picture of the *Typus Melancholicus* depicted by Hubertus Tellenbach. The COVID-19 lockdown (with no clues about when it would end) disrupted his orderly world, and he felt powerless, unable to put everything back in place, unable to provide for his family and unable to fulfill his financial duties (such as taxes, rent and pay for his staff).

Moving from Tellenbach description of the Typus Melancholicus, we can observe an ongoing evolution toward endogenous depression starting with a pre-melancholic phase. In this stage, three main elements can be described: includence, remanence, and despair.

The constellation of *includence* is a condition in which the attempt to maintain orderliness conflicts with the need to overcome it [15]. This creates stress in the personality structure of the patient who tries to go beyond his own limitations without changing his attitude toward orderliness and consciousness. During the lockdown, the loss of an organized daily routine and the lack of control over events puts the core stability of this personality structure at risk. B’s concerns were about his Shiatsu center and his customers. The changes imposed by the lockdown put.

the stability of the “immutable world” of the patient at serious risk, including his relationships and the affective bonds linked to his job. This new situation (the lockdown and the economic stress) undermined his psychological state that, hitherto, had been stable.

The constellation of *remanence* concerns the “danger of remaining behind regarding the subject’s own expectations” [15]. In such a condition, such as during a lockdown where time seems to be stuck, the Typus Melancholicus can not see a future. Everything is frozen in an empty present. During the lockdown, it was impossible for Mr. B to engage in his typical “nomothetic hyperactivity” and “hypernomic consciousness”: Mr. B was a very active person, working 12 h a day and always looking for some new life goals to accomplish. The lockdown clearly made it impossible for him to accomplish his unrealistic expectations. His situation overwhelmed Mr. B so that he felt “guilty” and “inauthentic” since he was not providing for his family as he felt that he should.

After these two constellations start to manifest, the patient begins the transition from a pre-morbid personality to a melancholy state, and perhaps an endogenous depression, as a result of the despair that is generated, leaving the patient feeling hopeless and helpless.

Another contribution to the role of pre-morbid personality on the development of psychiatric disorders was given by Ernst Kretschmer (1888–1964). The German psychiatrist focused on the exsisting correlation between biography and personality traits of patients later diagnosed as delusional with the introduction of the “sensitive delusion of reference” (sensitiver Beziehungswahn). He postulated that vulnerable and anancastic personality traits associated with real and repeated insults will first lead to a dysphoric and suspicious attitude, then to delusion-like ideas and, finally, to proper delusions. Kretschmer’s main purpose was to identify particular patterns that could eventually lead to delusional states. Kretschmer can be recognized as a pioneer, introducing the concept of “multidimensional psychiatry”, taking into account biological and biographical factors [16].

The pre-melancholic model described by Tellenbach might be a good model for understanding the psychological implications of the pandemic lockdown. The pandemic has elicited many psychiatric symptoms, especially for those with a pre-morbid personality structure which was in a fragile state before the pandemic began. The lockdown presents an opportunity to analyze the effects of socio-economic factors on the mental health of people with differing pre-morbid conditions during the “second wave” of affective disorders attendant upon the pandemic.

## Data Availability

This is a single-patient case report. Data sharing is not applicable to this article as no dataset were generated or analyzed in the present study.

## Abbreviations

COVID-19: Coronavirus disease 2019
SARS-CoV-2: Acute respiratory syndrome caused by the coronavirus 2
CAT: Computerized axial tomography
